# Supplementary material for: A Mixture of Essential Oils from Three Cretan Aromatic Plants Inhibits SARS-CoV-2 Proliferation: A Proof-of-Concept Intervention Study in Ambulatory Patients
Source: Diseases. 2023 Aug 9;11(3):105. doi: 10.3390/diseases11030105 (PMC10443288; doi:10.3390/diseases11030105)
Supplement: Supplementary file 1 [file diseases-11-00105-s001.zip › diseases-2534290-supplementary.pdf]

Supplement Tables

Table S1. Descriptive characteristics of the 69 patients in two Interventions & one Control groups.

			Groups		
			**Intervention**2020 (n=13)	**Intervention**2021/22 (n=25)	**Control**2021/22 (n=31)
			n (%)		
**Gender**	*males*		8 (61.5)	10 (40.0)	14 (45.2)
	females		5 (38.5)	15 (60.0)	17 (54.8)
**Age**, *years*	*mean age±s.d.*		36.4±11.7	39.5±13.5	42.1±16.1
**Family members**	*yes*		4 (30.8)	7 (28.0)	15 (48.4)
**Smokers**	*non*		8 (61.5)	14 (56.0)	13 (42.0)
	former		2 (15.4)	1 (4.0)	9 (29.0)
	current		3 (23.1)	10 (40.0)	9 (29.0)
**Morbidity** (at least one chronic disease)		*yes* [a]	4 (30.8)	10 (40.0)	9 (29.0)
**Recurrence of Covid-19** (in last six months)		*yes*	--	--	1 (3.2)
**Vaccination for SARS-CoV-2** (doses)		*none*	--	19 (50.0)	11 (35.5)
		one	--	5 (13.2)	3 (9.7)
		two	--	10 (26.3)	14 (45.2)
		three	--	4 (10.5)	3 (9.7)
**Administration/intake of medicinal or other compound for the symptoms** (before, at the point of or following inclusion to the study, additionally to CAPeo)		*painkillers*	3 (23.1)	13 (52.0)	19 (61.3)
		antibiotics	3 (23.1)	1 (4.0)	--

[a] *Includes nineteen different groups of diseases e.g. hypertension, diabetes mellitus, heart disease, cancer etc.*

Table S2. Mean changes in the number and severity of symptoms between two Interventions and one Control group at 7th and 14th days in relation to baseline (1st record day).

		Groups				
		**Intervention**2020	**Intervention**2021/22	**Control**2021/22		
Symptoms	Days of follow-up	mean (stand.dev.)			p-value	η^2
**Number**	**1st** (baseline)	4.3 (2.0)	4.8 (2.3)	4.0 (2.1)		
	**7th**	0.9 (1.7)	1.6 (1.9)	3.1 (3.1)		
	**Δ**-change (7th from 1st)	-3.4	-3.2	-0.9	0.002	0.15
	**14th**	0.3 (0.8)	0.6 (1.2)	1.1 (1.7)		
	**Δ**-change (14th from 7th)	-0.5	-1.0	-2.0	0.011	0.11
	**Δ**-change (14th from 1st)	-4.0	-4.2	-2.9	0.063	0.05
**Severity**	**1st** (baseline)	10.0 (5.5)	12.0 (6.2)	9.5 (5.2)		
	**7th**	2.0 (2.9)	4.2 (5.4)	8.1 (8.3)		
	**Δ**-change (7th from 1st)	-8.0	-7.8	-1.4	0.006	0.12
	**14th**	0.7 (1.8)	1.1 (2.5)	2.6 (4.3)		
	**Δ**-change (14th from 7th)	-1.3	-3.1	-5.6	0.043	0.07
	**Δ**-change (14th from 1st)	-9.3	-10.9	-6.9	0.033	0.07

Based on 12 topics and 9 general symptoms. The severity based on 5-points Likert scale as: 0=none, 1=very mild, 2=mild, 3=moderate and 4=severe. Scores of mean changes were extracted as summing-up all symptoms intensity. Comparisons of Δ-changes between the three groups were performed by using Kruskall-Wallis test.
